# From Breathlessness to Better Living: Transforming COPD Care with Home-based Pulmonary Rehabilitation

**DOI:** 10.5041/RMMJ.10543

**Published:** 2025-04-29

**Authors:** Abins Thozhuthinkal Kasim, Ravi Gaur, Nitesh Manohar Gonnade, Nagma Sheenam, Chinchu Kolakkanni, Sarankumar Ganesan, Adharshna Thangamalai Kannan

**Affiliations:** 1 Department of Physical Medicine and Rehabilitation, All India Institute of Medical Sciences, Jodhpur, Rajasthan, India; 2 Department of Physical Medicine and Rehabilitation, All India Institute of Medical Sciences, Nagpur, Maharashtra, India; 3 Department of Psychiatry National Institute of Mental Health and Neuro Sciences (NIMHANS), Bangalore, Karnataka, India

**Keywords:** Chronic obstructive pulmonary disease, disability, home-based rehabilitation, lung function, pulmonary disease, pulmonary rehabilitation, quality of life, WHODAS 2.0

## Abstract

**Background:**

Chronic respiratory diseases, such as chronic obstructive pulmonary disease (COPD), significantly impact patients’ quality of life by limiting physical function, mobility, and overall well-being. Pulmonary rehabilitation (PR), particularly home-based programs, has emerged as a vital non-pharmacological intervention to address these limitations. However, comprehensive assessments of the impact of home-based PR on both lung function and disability in COPD patients remain limited.

**Objective:**

This study aimed to evaluate the effectiveness of a 12-week home-based PR program on pulmonary function and disability in COPD patients, using pulmonary function tests (PFTs) and the World Health Organization Disability Assessment Schedule 2.0 (WHODAS 2.0) to assess outcomes across multiple domains.

**Methods:**

A prospective, single-arm pre–post interventional study was conducted among 62 COPD patients at All India Institute of Medical Sciences, Jodhpur. Participants completed a 12-week home-based PR program, which included endurance exercises, breathing techniques, and self-management education. Pulmonary function tests were conducted, and disability levels were assessed using WHODAS 2.0 at baseline and after completing the program.

**Results:**

Improvements were observed in pulmonary function, with forced vital capacity (FVC), and forced expiratory volume in one second (FEV1), showing substantial increases (FVC: 2.50±0.43 L to 2.85±0.59 L; FEV1: 1.53±0.33 L to 1.63±0.34 L; *P*<0.001). The WHODAS 2.0 scores demonstrated notable reductions in disability, particularly in the life activities and participation domains (*P*<0.001). Cognitive and self-care scores remained stable, while improvements in mobility were observed but not significant. Regression analysis revealed a strong negative correlation between increases in FVC and reductions in WHODAS 2.0 total scores (*r*=−0.65), highlighting FVC as a key predictor of disability reduction.

**Conclusion:**

The 12-week home-based PR program improved lung function and reduced disability in COPD patients. These findings support the role of home-based PR as a viable, patient-centered alternative to traditional rehabilitation, addressing both physical and social dimensions of health. Future research should focus on long-term outcomes, the potential for broader implementation, and expanding access to underserved populations.

## INTRODUCTION

Chronic respiratory diseases, such as chronic obstructive pulmonary disease (COPD), significantly impact quality of life, limiting physical function, mobility, and overall well-being.^1^,^2^ These impairments are primarily due to the decline in pulmonary function, characterized by airflow limitation, hyperinflation, and impaired gas exchange, leading to reduced exercise capacity and increased breathlessness.^3^ Standard management for these conditions has traditionally focused on pharmacological interventions, such as bronchodilators, corticosteroids, and oxygen therapy. While these treatments alleviate symptoms and prevent exacerbations, they often fail to comprehensively address the physical, psychological, and functional limitations experienced by patients in their daily lives.^4^

In recent years, pulmonary rehabilitation (PR), particularly home-based programs, has gained attention as a vital non-pharmacological intervention for managing chronic respiratory diseases. Home-based PR programs typically combine supervised exercise training, disease management education, and personalized self-management strategies, all delivered within the convenience of the patient’s home.^5^ This approach provides significant advantages, such as greater accessibility for those who may struggle to attend clinic-based programs due to mobility issues, transportation limitations, or geographical barriers. Additionally, home-based PR has been associated with improved adherence, as patients can integrate rehabilitation activities into their daily routines more easily, potentially leading to better long-term outcomes.^6^,^7^

Accurate and holistic evaluation of disability in individuals with chronic respiratory conditions is crucial for tailoring treatment plans, optimizing rehabilitation efforts, and ensuring efficient allocation of healthcare resources. Although spirometry is the cornerstone diagnostic tool for identifying and quantifying airflow limitation, it primarily reflects lung mechanics and does not capture the broader impact of respiratory diseases on daily functioning, social participation, or psychological well-being.^8^–^11^ To address this gap, several patient-reported outcome measures have been developed, offering a more comprehensive assessment of a patient’s functional capacity and overall disability. Among these tools, the World Health Organization Disability Assessment Schedule 2.0 (WHODAS 2.0) stands out for its ability to assess multiple dimensions of disability, including physical, social, and emotional domains, offering a holistic perspective on how chronic conditions affect patients’ lives.^12^ Research has demonstrated significant correlations between WHODAS 2.0 scores and other established disability metrics, as well as exercise capacity measures such as the six-minute walk test and healthcare utilization patterns, further supporting its validity and utility in clinical practice.^13^–^16^

This study aims to explore the impact of home-based PR on lung function and disability in patients with chronic respiratory diseases. Specifically, it hypothesizes that participation in home-based PR will lead to significant improvements in lung function, as evidenced by pulmonary function test (PFT) results, and reductions in disability, as assessed by WHODAS 2.0. By investigating these outcomes, the study highlights the growing importance of home-based care as a viable alternative to conventional rehabilitation methods and underscores the need for comprehensive, multidimensional disability assessment tools to enhance patient care and outcomes.

## PATIENTS AND METHODS

This prospective, single-arm, pre–post interventional study aimed to evaluate the impact of a 12-week home-based PR program on pulmonary function and disability in patients with COPD. The study was conducted at All India Institute of Medical Sciences (AIIMS), Jodhpur, following approval from the Institute Ethics Committee (Approval No. AIIMS/IEC/2021/3523). Written informed consent was obtained from all participants after they had been informed of the study objectives, procedures, and potential risks.

### Study Population

Participants were recruited from outpatient clinics at AIIMS Jodhpur. The inclusion criteria required patients to be between 40 and 70 years of age, diagnosed with COPD based on the Global Initiative for Chronic Obstructive Lung Disease (GOLD) guidelines (stages 1–3),^4^ and stable for at least one month without recent exacerbations or hospitalizations. Patients capable of following home-based PR and performing physical exercises were included. Exclusion criteria were: recent hospitalization (within the past month), heart disease limiting physical function, contraindications to exercise (e.g. unstable angina, severe musculoskeletal issues), bedbound or moribund patients, pre-existing psychiatric illnesses or cognitive impairments, patients on long-term oxygen therapy, active smokers, and individuals unwilling to quit smoking during the study period.

### Baseline Assessments

Participants underwent baseline assessments, including a comprehensive medical history review, physical examination, and PFTs measured by spirometry. The medical history captured COPD duration, comorbidities, medications, smoking history, and any prior pulmonary rehabilitation. Physical examination measured vital signs such as blood pressure, heart rate, oxygen saturation, and respiratory rate. Spirometry tests recorded forced vital capacity (FVC), forced expiratory volume in 1 second (FEV1), and the FEV1/FVC ratio. Each participant performed three trials, and the highest values were recorded.

Disability was assessed using the WHODAS 2.0, which evaluates disability across six domains: cognition, mobility, self-care, getting along, life activities, and participation in society. Researchers assisted participants as needed to complete the questionnaire.

### Home-based Pulmonary Rehabilitation Program

The 12-week home-based PR program was designed based on American Thoracic Society/European Respiratory Society guidelines.^17^,^18^

At the start of the program, patients received in-person training sessions at the hospital where Rehab team members demonstrated each exercise, including endurance activities, breathing techniques, and upper limb strengthening exercises. Patients were provided with written and illustrated instructions as take-home materials. While the exercises were not supervised in real-time, monthly telephonic follow-ups served to monitor progress, resolve difficulties, and reinforce techniques. Adherence was self-reported by patients during these follow-up calls.

The program included:

#### Exercise training

Endurance exercises such as walking or cycling, which were progressively increased from 10–15 minutes to 30 minutes per session by the end of the program. Thoracoabdominal exercises, focusing on diaphragmatic breathing, were included to strengthen respiratory muscles. Upper limb strengthening exercises, using light weights or resistance bands, were prescribed to improve physical conditioning.

Patients were asked to perform the above exercises three times per week, with each session lasting about 60 minutes.

#### Breathing techniques

Controlled breathing techniques, such as pursed-lip breathing and diaphragmatic breathing, were taught to enhance respiratory efficiency and reduce dyspnea.

#### Education and self-management

Patients received education on COPD pathophysiology, exacerbation recognition, medication adherence, smoking cessation, and nutritional advice. Symptom management strategies for breathlessness, coughing, and fatigue were emphasized.

#### Monitoring and follow-up

Monthly follow-up interviews by telephone covered exercise adherence, symptom management, and overall well-being.

### Follow-up Assessments

At the end of the 12-week PR program, participants returned for follow-up assessments, including repeat spirometry to measure lung function changes (FVC, FEV1, FEV1/FVC ratio) and a re-administration of the WHODAS 2.0 questionnaire to assess changes in disability status. When necessary, participants were contacted for further follow-up.

### Statistical Analysis

Statistical analyses were conducted using IBM SPSS version 22. Descriptive statistics, including means and standard deviations, were used to summarize participant characteristics, PFT results, and WHODAS 2.0 scores. Paired *t*-tests were used to compare pre- and post-program PFT parameters, while one-way repeated measures ANOVA was applied to assess changes in disability scores over time. The Greenhouse–Geisser correction was applied where necessary. A *P*-value of <0.05 was considered statistically significant.

Changes in PFT were calculated by analyzing the changes observed in the different variables between baseline and 12 weeks. A similar analysis was performed for the WHODAS 2.0 total score. Correlation coefficients were calculated to assess associations between changes in pulmonary function tests and WHODAS 2.0 scores. Strength of correlations was interpreted according to Evans, who classifies correlations as very weak (<0.20), weak (0.20–0.39), moderate (0.40–0.59), strong (0.60–0.79), and very strong (>0.80). Correlations were considered statistically significant if the *P*-value was <0.05, indicating that the observed relationships were unlikely to be due to chance.^19^

In the absence of directly comparable data in the literature, a convenience sampling method was utilized to recruit participants from the outpatient clinics at AIIMS Jodhpur, based on their availability and willingness to participate.

### Ethical Considerations

The study adhered to the ethical principles of the Declaration of Helsinki. All participants provided informed consent, and confidentiality of data was maintained throughout the study. Ethical approval was granted by the Institutional Ethics Committee of AIIMS Jodhpur (Approval No. AIIMS/IEC/2021/3523).

## RESULTS

### Patient Population

During the study period, 88 patients were recruited for the 12-week home-based PR program. Of these, 26 patients were lost to follow-up, resulting in 62 patients completing the full 3-month follow-up period after the PR program. The mean age of the participants was 57.2±9.9 years. The demographic details of the participants are summarized in Table 1.

**Table 1:** Demographic Details.

Parameter	Patients (*n*=88)	Percentage (%)
Age (mean±SD)	57.2±9.9 years	--
Sex, *n* (%)		
Male	56	64.0
Female	32	36.0
BMI (mean±SD)	22.10±3.3	

Hb value (mean±SD)	13.82±1.1	

Educational level		
Primary education	44	50.0
Middle school	24	11.4
High school	15	17.1
Undergraduate degree	4	4.5
Graduate degree	1	1.1

Living situation		
Staying with family	86	97.7
Alone	2	2.3

Dietary habits		
Vegetarian	51	58.0
Non-vegetarian	37	42.1

Length of time with disease (years)		
Less than 1	22	25
1 to 3	34	38.6
3 to 5	18	20.5
More than 5	14	15.9

Motivation for participation		
No motivation	14	15.9
Yes, self-motivated	15	17.1
Yes, family encouragement	11	12.5
Yes, doctor’s recommendation	2	2.2
Yes, social influence	4	4.6
Yes, health benefits	42	47.7

Marital status		
Married	88	100
Unmarried	0	0

Previous pulmonary rehabilitation (Yes/No)		
Yes	14	15.9
No	74	84.2

Work status		
Working	26	29.5
Stopped due to illness	31	35.2
Changed job due to illness	9	10.2
Already retired from job	22	25

Previous lung surgeries		
Yes	2	2.2
No	86	97.8

### Spirometry Results

Significant improvements were observed in the PFT parameters after the completion of the PR program. Specifically, FVC and FEV1 improved (*P*<0.001). Detailed changes in the mean PFT values during the study period are presented in Table 2.

**Table 2:** Mean Pulmonary Function Test (PFT) Values Before and After Pulmonary Rehabilitation (PR).

Parameter	Before PR (Mean±SD)	After PR (Mean±SD)	*P*-Value
FVC (L)	2.50±0.43	2.85±0.59	<0.001
FEV1 (L)	1.53±0.33	1.63±0.34	<0.001
FEV1%	59.34±12.64	63.02±12.74	<0.001
FEV1/FVC	0.62±0.10	0.71±0.63	0.21

FEV1, forced expiratory volume in one second; FVC, forced vital capacity.

### Disability Scores: WHODAS 2.0 Analysis

The disability levels of the participants were assessed on a monthly basis using the WHODAS 2.0 across six domains: cognition, mobility, self-care, getting along, life activities, and participation in society.

Participation in home-based PR was associated with a significant reduction in the disability score, as assessed by WHODAS 2.0 (Table 3). Improvement was observed in the getting along, life activities, and participation domains. No improvements were observed in the cognition and self-care domains. Slight improvement in mobility did not prove significant.

**Table 3:** WHODAS 2.0 Scores during Study Duration.

Domain	Month 0 (Mean±SD)	Month 1 (Mean±SD)	Month 2 (Mean±SD)	Month 3 (Mean±SD)	*P*-Value
Cognition	4.6±1.9	4.5±1.9	4.5±1.9	4.6±1.9	>0.05
Mobility	9.4±1.5	9.4±1.5	9.3±1.6	8.6±1.7	>0.05
Self-care	4.7±1.3	4.4±1.2	4.6±1.2	4.4±1.2	>0.05
Getting along	8.8±1.1	8.2±1.1	8.6±1.1	8.2±1.1	<0.001
Life activities	17.9±5.1	17.9±5.1	17.7±5.1	16.7±5.0	<0.001
Participation	15.2±1.8	15.2±1.8	14.7±1.8	14.4±1.8	<0.001
Total score	60.5±6.7	60.5±6.7	59.4±6.9	56.8±6.8	<0.001

### Association Between Pulmonary Function Parameter Changes and Change in WHODAS 2.0

A multiple linear regression analysis was conducted to evaluate the association between changes in pulmonary function parameters (FVC and FEV1) and the change in WHODAS 2.0 total scores, which reflect disability and quality of life improvements. Additionally, Pearson correlation coefficients were calculated to assess the strength of these associations.

Regression analysis revealed that both FVC and FEV1 had significant negative coefficients, indicating that increases in these measures were associated with reductions in the WHODAS 2.0 total scores, reflecting improvements in disability and functional outcomes. Specifically, FVC had a coefficient of −30.0, meaning the WHODAS 2.0 score decreased by 30 points for each unit increase in FVC, with a moderate to strong Pearson correlation of −0.7. In contrast, FEV1 had a larger negative coefficient of −77.9, indicating substantial reductions in WHODAS 2.0 scores with its increase. However, the weaker Pearson correlation of −0.2 indicated a less consistent relationship. Overall, these findings underscore the significant role of FVC in predicting improvements in disability, while FEV1 plays a noteworthy but less consistent role.

## DISCUSSION

This study provides compelling evidence for the significant benefits of a 12-week home-based PR program on both pulmonary function and disability reduction in patients with chronic respiratory conditions, particularly COPD. The results demonstrated substantial improvements in key lung function parameters, notably FVC and FEV1, highlighting the effectiveness of home-based PR in improving respiratory function. These findings align with prior research by Priya et al.^20^ and Pradella et al.,^21^ who similarly reported improvements in lung function following PR interventions. The results are also supported by the studies of Kjærgaard et al.,^22^ Zhang et al.,^23^ and Naseer et al.,^24^ highlighting that short-term PR programs are effective in promoting better lung health and overall quality of life in COPD patients.

The home-based PR program offers a patient-centered approach that enhances engagement and adherence by addressing barriers such as accessibility, mobility limitations, transportation challenges, and geographic constraints. By enabling rehabilitation in a familiar environment, adherence rates are markedly improved and patients are able to integrate exercises and self-management techniques into their daily routines. Additionally, emotional and practical support from family members further boosts motivation and adherence, fostering a sense of autonomy and control over health. These advantages are also supported by the studies of Pradella et al.^21^ Overall, a home-based personalized PR approach offers distinct advantages over traditional hospital-based programs, ultimately leading to improved adherence and patient outcomes.^5^

Weekly follow-up by telephone provided personalized feedback, motivation, and solutions to challenges, ensuring accountability and strengthening the patient–provider connection. The program’s individualized approach, tailoring exercises and breathing techniques to patient-specific needs, increased its relevance and feasibility. The accessibility, flexibility, emotional support, and consistent engagement factors led to significant improvements in pulmonary function and reduced disability, especially in life activities and participation.^24^,^25^

One of the strengths of this study lies in its use of WHODAS 2.0, which provided a comprehensive assessment of disability across multiple domains. The significant reductions in WHODAS 2.0 total scores, particularly in life activities and participation domains, reflect a marked improvement in patients’ ability to manage daily tasks and engage socially. These improvements highlight the broader benefits of PR beyond just respiratory function, addressing key aspects of everyday life. These findings are consistent with the studies of da Silva e Silva et al.^16^ and Zacarias et al.,^12^ which also demonstrated the positive impact of PR on disability reduction in COPD patients.^26^ Furthermore, the feasibility and cost-effectiveness of using WHODAS 2.0 in COPD patients is supported by Potcovaru et al.,^27^ reaffirming the tool’s utility in clinical practice.

The stable cognitive scores observed in this study likely reflect the limited direct impact of the home-based PR program on cognitive function. While physical exercise and improved oxygenation can have neuroprotective benefits, the program’s focus was primarily on physical rehabilitation and respiratory efficiency, with no specific interventions targeting cognitive stimulation. This stability, however, is a positive finding, indicating that the PR program did not negatively affect cognitive function in an older population at risk for cognitive decline. Future iterations of home-based PR could incorporate cognitive exercises, such as problem-solving tasks or memory training, to address this domain and enhance holistic rehabilitation.

The less significant improvements in mobility may stem from the complex interplay of factors contributing to mobility limitations in COPD patients. Reduced exercise tolerance, balance impairments, and lower extremity muscle deconditioning are common in this population and may not be fully addressed by endurance and thoracoabdominal exercises alone. Additionally, severe dyspnea during physical activity could have limited the intensity or consistency of mobility-focused exercises for some participants.^28^,^29^

These findings suggest that home-based PR programs should be tailored to include targeted interventions for mobility, such as resistance training for lower limb muscles, balance exercises, and graduated walking programs. Incorporating assistive devices, if needed, and strategies to manage dyspnea during exertion could further enhance mobility outcomes. Addressing these aspects can ensure that PR programs comprehensively improve all domains of disability, including mobility, thereby maximizing their overall effectiveness.

The strong negative correlation between improvements in FVC and the lower WHODAS 2.0 scores further emphasizes the link between better lung function and reduced disability. These data also highlight the potential role played by lung function improvement in reducing disability. However, the weaker association with FEV1 suggests that other factors, such as exercise tolerance, dyspnea management, or psychosocial improvements, may play an equally important role in influencing disability outcomes. On the other hand, while FEV1 also showed an association with disability reduction, the correlation was weaker, suggesting that FVC might be a more consistent indicator of functional outcomes. These findings suggest that PR programs should prioritize not only lung function improvement, but also a broader assessment of disability to capture the holistic benefits of rehabilitation.^30^

This study’s single-arm design and lack of a control group limited the ability to attribute observed improvements solely to the home-based PR program. Without a comparison group, external factors such as natural disease progression or other influences may have contributed to the results. Furthermore, the absence of long-term follow-up restricts insights into the sustained effects of PR on lung function, disability, and adherence over time.

The dropout rate of 26 patients suggests challenges in maintaining adherence to home-based programs, particularly over extended periods. Future studies should address these issues by integrating telemedicine and digital health tools to enhance program reach and effectiveness. Cognitive assessments should also be incorporated to provide a holistic understanding of PR’s impact on physical, psychological, and cognitive health in COPD patients. These efforts will ensure a more comprehensive evaluation of PR programs and their outcomes.

The study’s findings, while promising, may not fully generalize to the broader COPD population due to the lack of diversity in baseline characteristics and healthcare access among participants. Extended follow-up periods are essential to validate the durability of benefits, including sustained improvements in lung function, disability reduction, and quality of life. Stratified analyses based on COPD severity (e.g. GOLD stages) and other patient characteristics could further refine personalized rehabilitation strategies.

Despite these limitations, the study strongly supports the transformative potential of home-based PR programs. These programs improve lung function, reduce disability, and enhance quality of life by addressing physical, social, and functional health dimensions. The use of WHODAS 2.0 as a comprehensive disability assessment tool underscores the importance of a biopsychosocial approach to COPD management, ensuring that both physical and social aspects of patient care are prioritized.

To optimize the design and implementation of home-based PR programs, research should explore additional domains, including cognitive and psychosocial outcomes. Tailored mobility-specific strategies can address areas with less significant improvement, ensuring comprehensive care. By advancing the evidence base and refining program designs, home-based PR has the potential to become a sustainable, accessible, and effective rehabilitation model for COPD patients worldwide.

## Abbreviations

COPD: chronic obstructive pulmonary disease
FEV1: forced expiratory volume in one second
FVC: forced vital capacity
GOLD: Global Initiative for Chronic Obstructive Lung Disease
PFT(s): pulmonary function test(s)
PR: pulmonary rehabilitation
WHODAS 2.0: World Health Organization Disability Assessment Schedule 2.0
